# Invasive Community-Acquired Methicillin-Resistant Staphylococcus aureus (MRSA) Infection in Children: A Report of Five Cases and Literature Review

**DOI:** 10.7759/cureus.37974

**Published:** 2023-04-22

**Authors:** Dhuha A Alidrisi, Waad Alharthi, Tariq Alfawaz

**Affiliations:** 1 Pediatric Infectious Diseases, Security Forces Hospital Makkah, Makkah, SAU; 2 Pediatric Infectious Diseases, King Fahad Specialist Hospital, Dammam, SAU; 3 Pediatric Infectious Diseases, King Fahad Medical City, Riyadh, SAU

**Keywords:** pediatric, methicillin resistant staphylococcus aureus (mrsa), community acquired infection, consequences, invasive mrsa, children

## Abstract

*Staphylococcus aureus* developed resistance against most antibiotics; the most known resistant form is methicillin-resistant *Staphylococcus aureus* (MRSA), which can be acquired either from healthcare facilities or the community. The prevalence of hospital-acquired MRSA is higher than community-acquired MRSA (CA-MRSA). CA-MRSA has become an emerging infection and has been increasingly reported recently. Usually, CA-MRSA presents with skin and soft tissue infection but can cause severe invasive infection with significant morbidity. Invasive CA-MRSA needs rapid and aggressive treatment to prevent complications. For MRSA bacteremia that is persistent despite appropriate treatment, the possibility of metastatic invasive infection should be thought of. In this case series, we describe five pediatric cases of different age groups that had different presentations for invasive CA-MRSA infection. This report aims to highlight that physicians should be aware of the growing role of CA-MRSA in pediatrics; they should be meticulous in treating patients with CA-MRSA, and be aware of the complications of this disease and the appropriate empiric and target antibiotics regimen for such infections.

## Introduction

Invasive methicillin-resistant *Staphylococcus aureus* (MRSA) infection in children is associated with high morbidity and mortality [1-2]. MRSA causes a spectrum of diseases, most commonly causing skin and soft tissue infections such as cellulitis and abscess, and life-threatening systemic infections like infective endocarditis, osteoarticular infection, and necrotizing pneumonia in association with bacteremia have been reported [3,4]. Around 25-30% of patients with *S. aureus* bacteremia will develop invasive complications, with ﻿an associated mortality rate of about 30% [5-6]. Some virulent strains of community-acquired MRSA (CA-MRSA) produce the toxin Panton-Valentine leucocidin (PVL), which can lead to more invasive disease [7-9].

A previous study conducted in Saudi Arabia demonstrated an increase in the prevalence of CA-MRSA infection ranging between 23-42% in different regions in Saudi Arabia [10]. Similar series have earlier described cases of CA-MRSA bacteremia complicated with osteoarticular infection, necrotizing pneumonia, and infective endocarditis [11]. Although there are similar case reports and case series on invasive CA-MRSA infection, our series aims to highlight the wide spectrum of invasive CA-MRSA disease and to demonstrate different clinical outcomes in five cases with invasive disease.

## Case presentation

Five cases are been described in this report. The summary of the cases are given in Table 1.

**Table 1:** Clinical summary of cases presented with invasive CA-MRSA disease CA-MRSA: community-acquired methicillin-resistant *Staphylococcus aureus*

Age	Gender	Infection site	Treatment	Outcome
11 years	Male	Left foot chronic osteomyelitis	Vancomycin then switched to PO linezolid	Improved
6 weeks	Male	Bloodstream infection and multiple-site osteomyelitis	Vancomycin followed by IV clindamycin	Improved
12 years	Female	Bloodstream infection, infective endocarditis with cerebral abscesses	Vancomycin with rifampin and gentamicin	Improved
1 month	Male	Bloodstream infection, with presumed infective endocarditis	Vancomycin and rifampin	Improved
9 years	Female	Bloodstream infection, with complicated pneumonia	Vancomycin with gentamicin	Improved

Case 1

An 11-year-old male child presented with acute fever, swelling in the left foot swelling, and limping preceded by trauma, with elevated inflammatory markers. Initial partial debridement done at the primary hospital revealed MRSA from the pus, which was sensitive only to vancomycin, which was started with an interrupted course for 10 days, and then referred to us with persistent local symptoms but with no fever in addition to elevated inflammatory markers.

**Figure 1:**
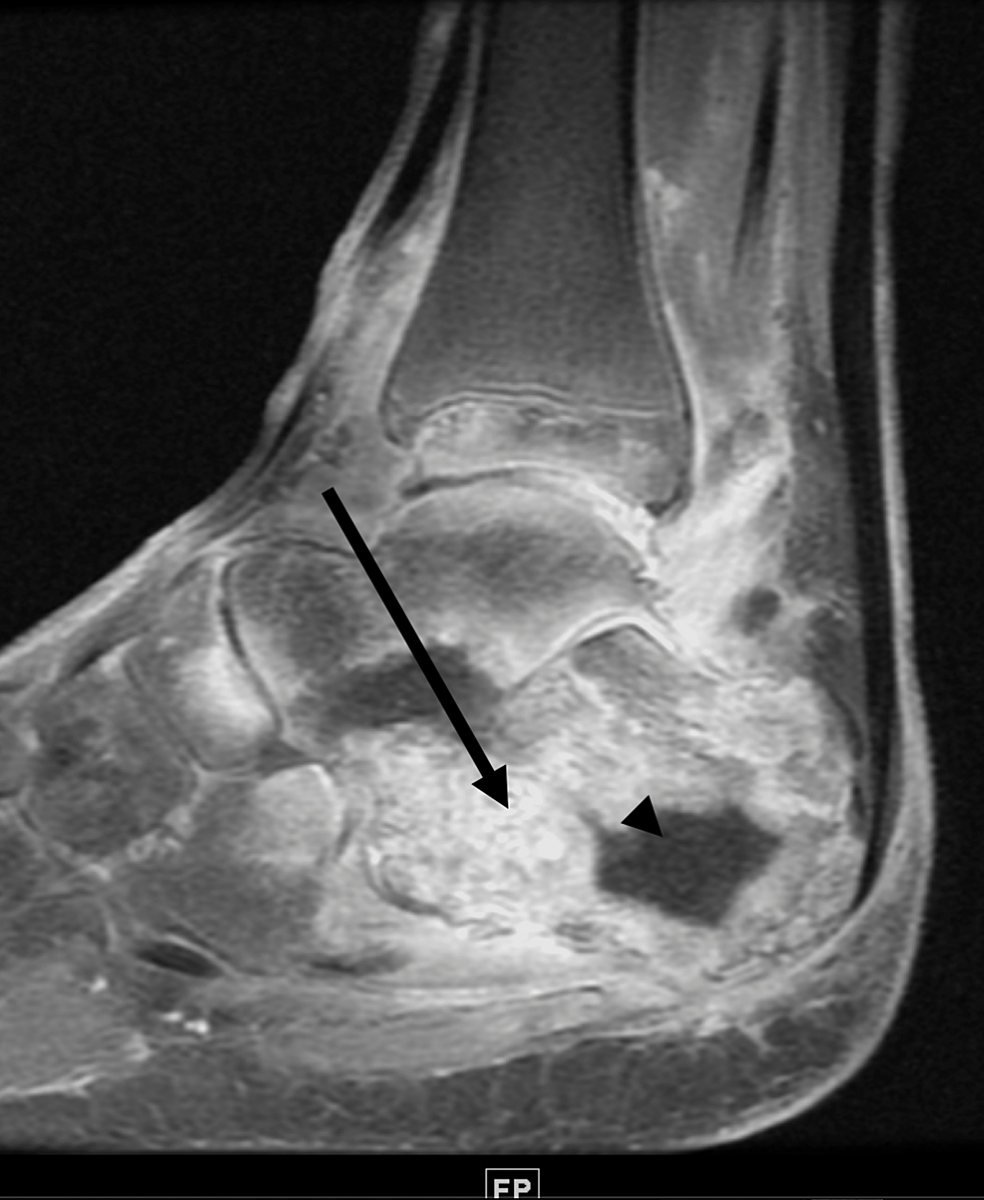
Contrast-enhanced MRI of left ankle joint in Case 1 demonstrated intense calcaneal bone marrow edema and enhancement on post-contrast administration (long arrow) and a focal area of non-enhancement suggestive of necrotic bone marrow (arrow head).

MRI of the ankle and foot demonstrated extensive destructive calcaneal changes with the destruction of the subtalar joint representing advanced osteomyelitis with bone necrosis and extensive soft tissue edema/cellulitis with associated septic arthritis (Figure 1). The pus was aspirated from calcaneal bone and the culture grew MRSA again. The patient was treated with vancomycin for six weeks giving good symptom resolution and improvement in inflammatory markers. He was discharged on oral linezolid and rifampin; to be treated for chronic osteomyelitis.

Case 2

**Figure 2:**
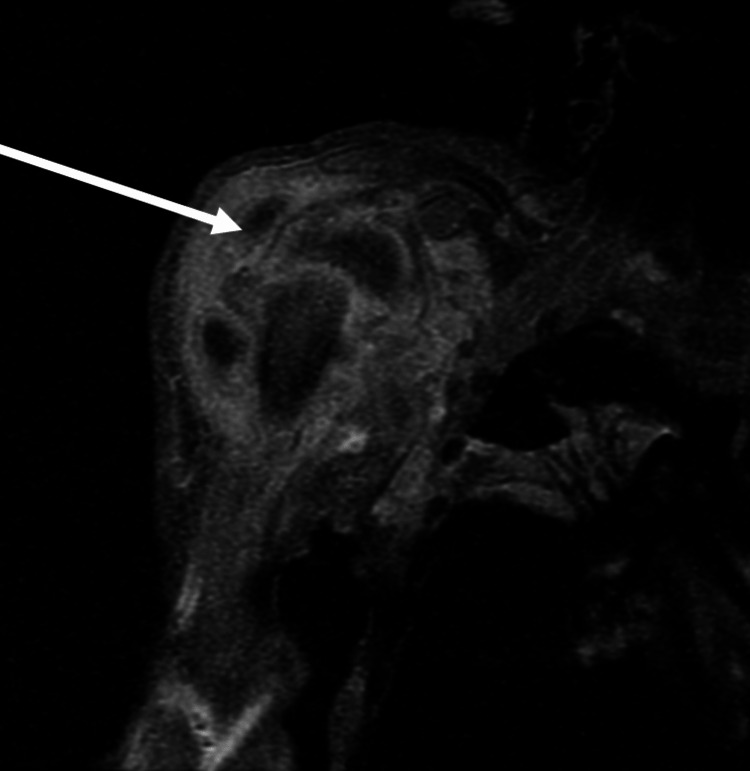
Post-contrast image of MRI right shoulder in Case 2 demonstrated multiple collections in the subacromial subdeltoid bursa with intense surrounding bone marrow edema (long arrow).

**Figure 3:**
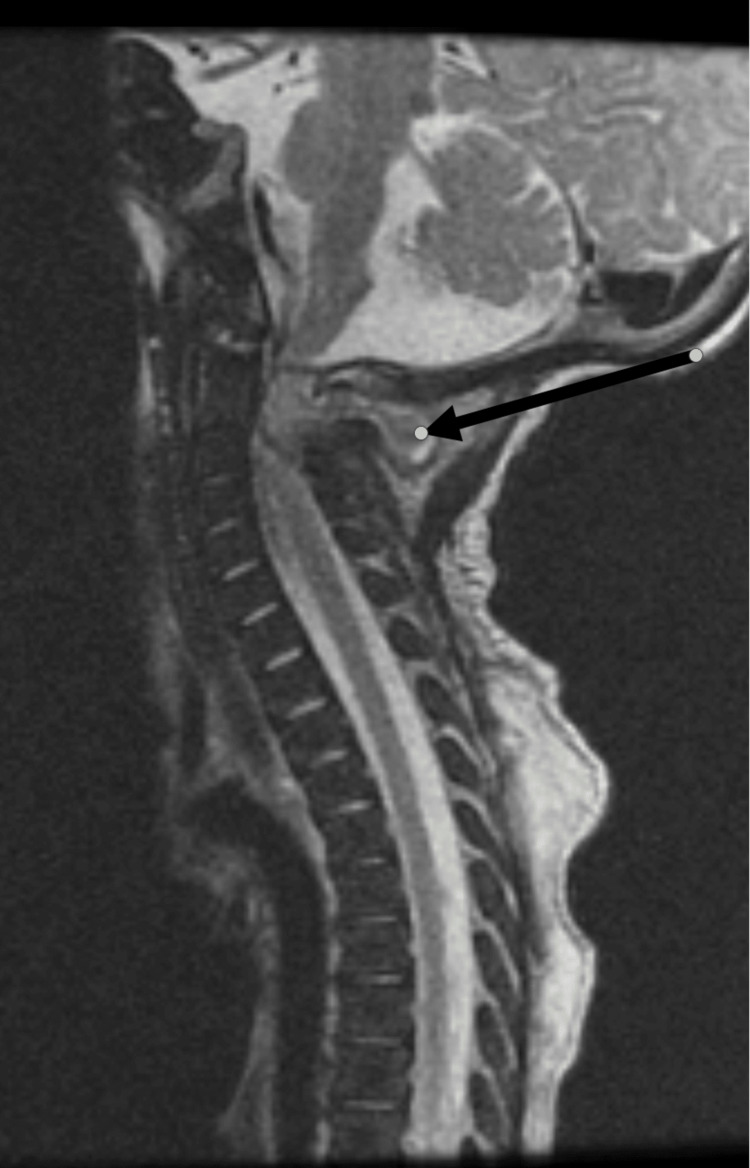
Sagittal T2 weighted image of the upper cervical spine in Case 2 demonstrated heterogenous collection in the craniocervical junction with intraspinal extension (long arrow).

A six-week-old male child presented with a history of swelling of the right shoulder and right ankle with limitation of movement for two weeks and a history of acute fever. Initial workup showed WBC 34.5 x10^9^/L with 68% neutrophils, erythrocyte sedimentation rate (ESR) of 97 mm/h, and C-reactive protein (CRP) of 299 mg/dl. Blood culture grew CA-MRSA as it was sensitive to clindamycin and vancomycin. Shoulder joint aspiration was done, which grew MRSA. Surface swabs were also positive for MRSA. MRI of the right shoulder showed osteomyelitis involving the upper metaphysis of the right hummers extending to the epiphysis with a picture of pyomyositis at the deltoid area (Figure 2). MRI whole spine showed an upper posterior cervical lesion most likely representing an abscess with a smaller intraspinal epidural component causing a mass effect on the adjacent spinal cord (Figure 3). The patient was treated with intravenous antibiotics over the whole illness course (six weeks) with documented clinical as well as laboratory improvement. The initial workup did not demonstrate any immune defect.

Case 3

**Figure 4:**
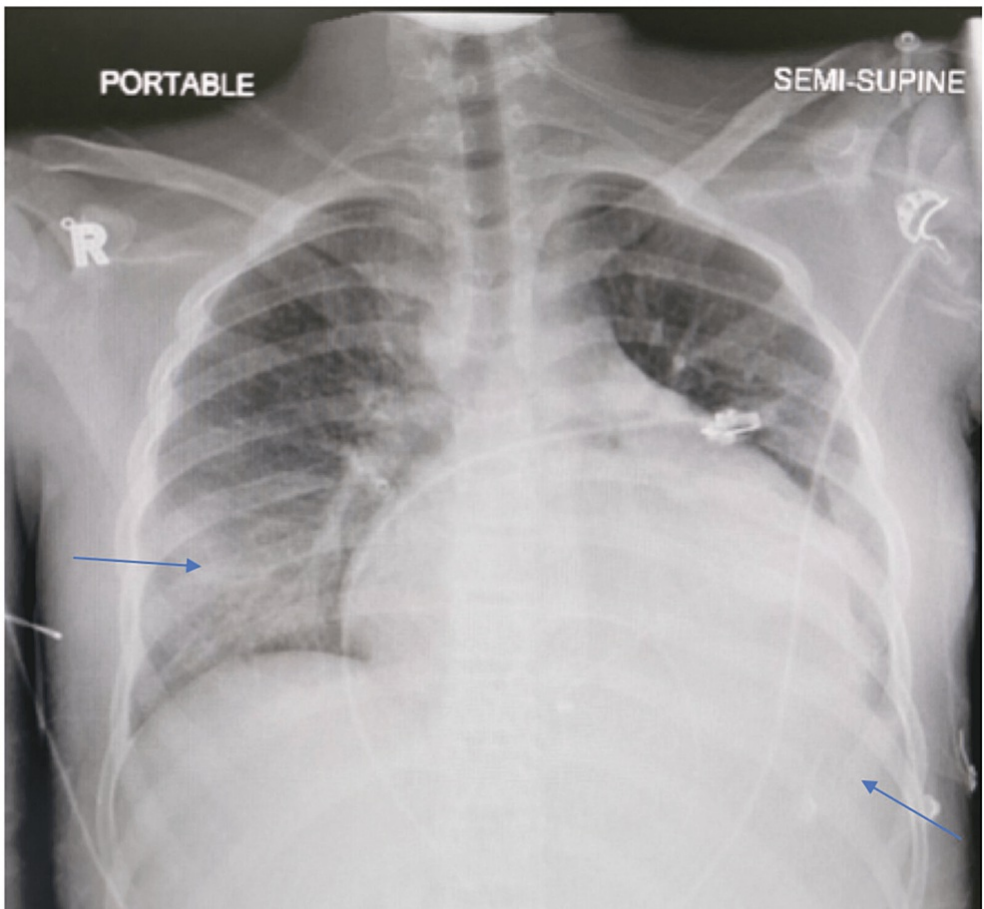
Chest X-ray in Case 3 showed bilateral lower lobe atelectasis with left-sided pleural effusion.

**Figure 5:**
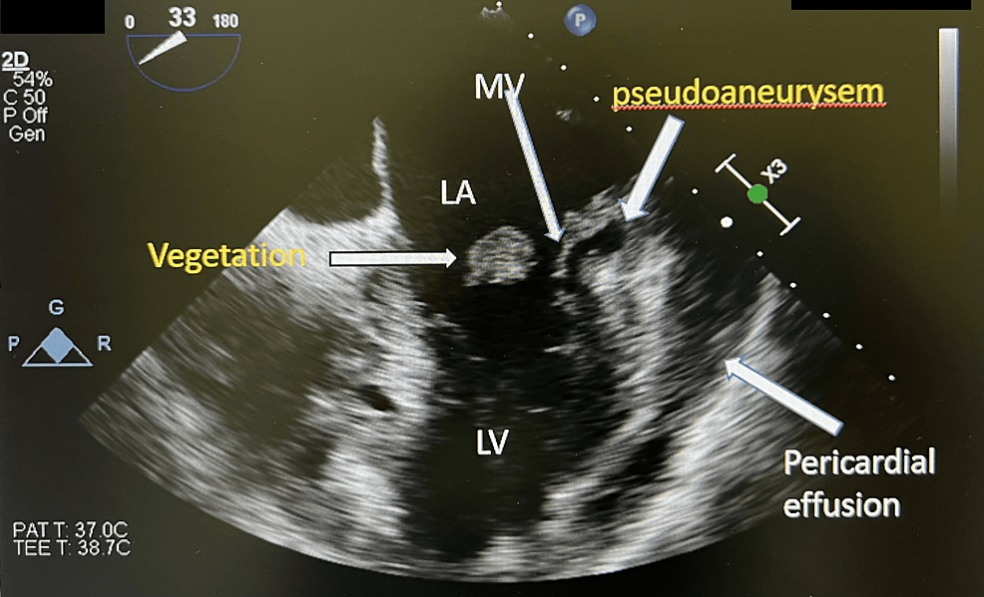
Echocardiography in Case 3 revealed vegetation with evidance of pseudoaneurysem.

**Figure 6:**
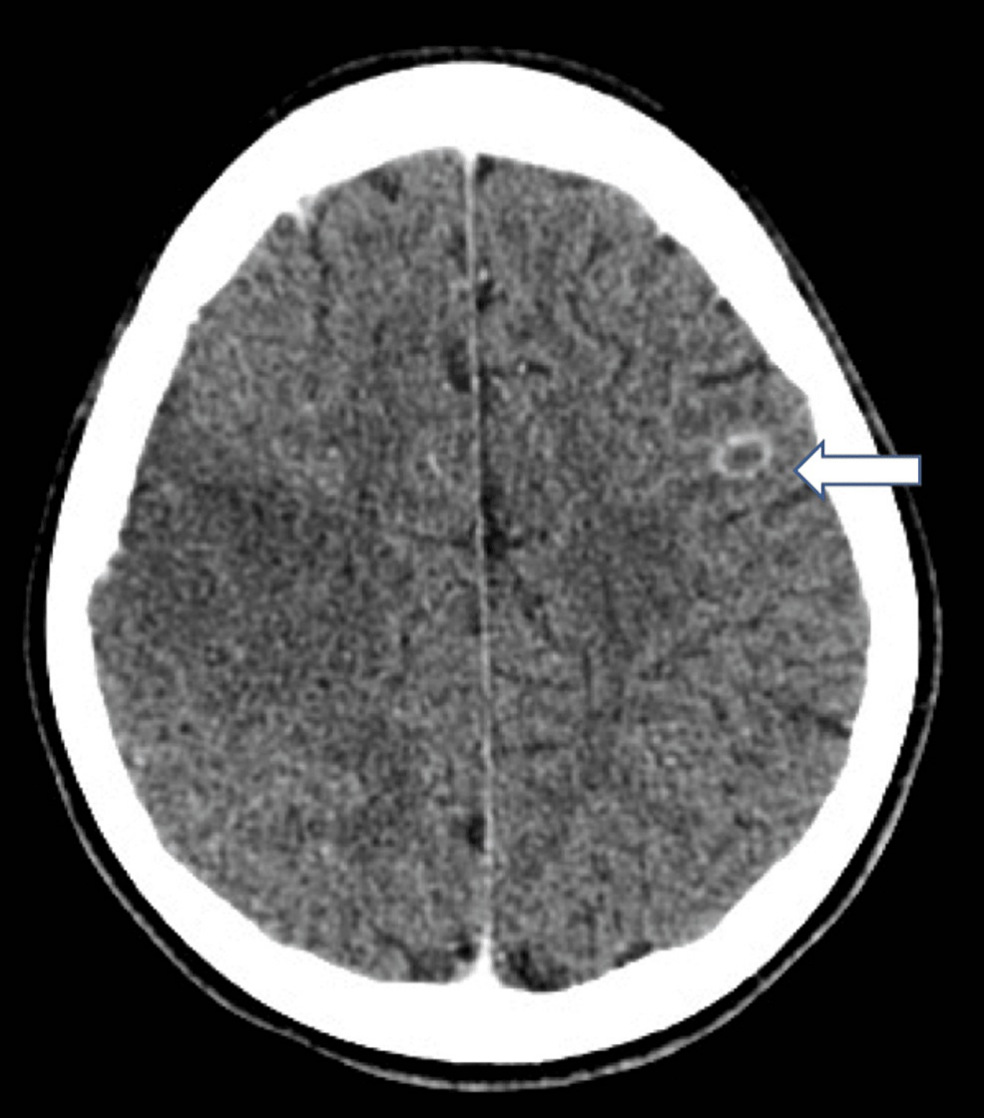
CT brain in Case 3 showed a ring-enhancing lesion in the left frontal lobe suggesting abscesses.

A 12-year-old female child, not known to have any medical illness before, presented with fever, headache, and vomiting for three days with altered mental status. On examination, she looked septic and febrile, her level of consciousness was decreased but she was arousable, she had neck stiffness, but other meningeal signs were not elicited. The patient was started on vancomycin but the initial workup was unremarkable with negative blood culture, so the antibiotic was discontinued. The patient deteriorated rapidly and spiked a fever again with tachypnea. Pan systolic murmur was heard on examination. X-ray chest showed lung infiltration with bilateral effusion (Figure 4), and echocardiography revealed two masses in the left ventricles (Figure 5). CT brain revealed a ring-enhancing lesion in the left frontal lobe suggesting brain abscesses (Figure 6). She was diagnosed with infective endocarditis with cerebral abscesses and received IV vancomycin, cefepime, and ampicillin without any improvement. Blood culture then grew MRSA. Gentamicin plus rifampin was added to vancomycin and other antibiotics were discontinued.

**Figure 7:**
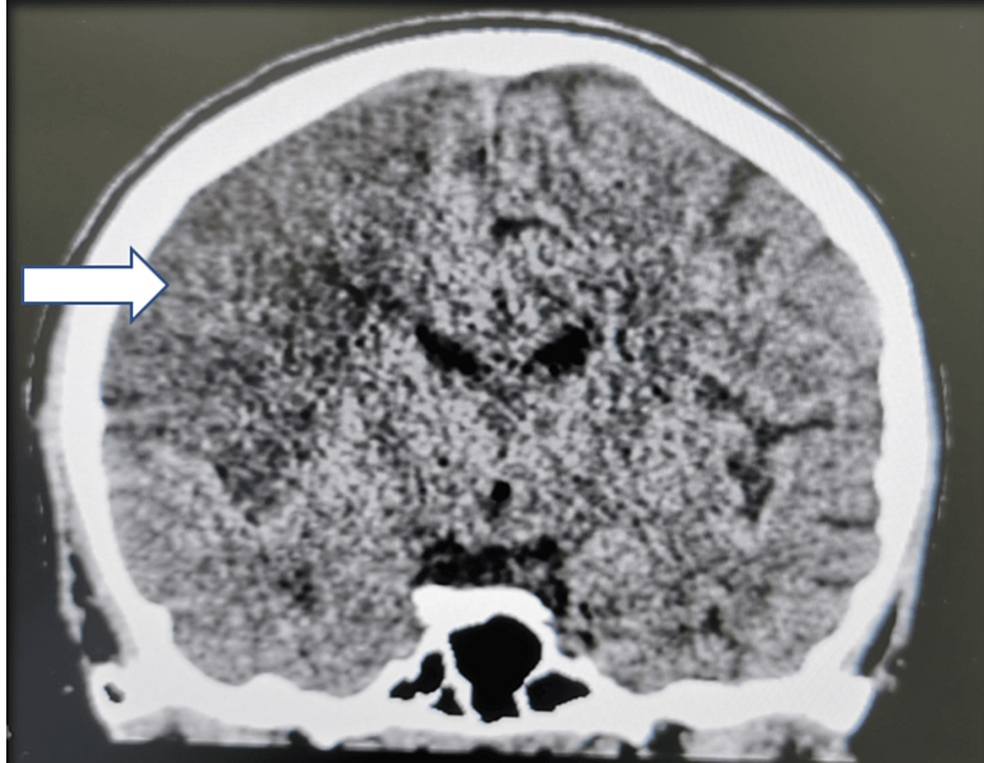
MRI brain in Case 3 showed that the patient developed right ischemic infarction of middle cerebral artery.

The next day the patient developed a right ischemic infarction of the middle cerebral artery (Figure 7), then underwent surgery to remove the cardiac vegetation. She received vancomycin and rifampin for six weeks and gentamicin for seven days. At the end of therapy, echocardiography showed complete resolution of the cardiac vegetation, and repeated CT brain showed resolution of brain abscess. Clinically she was stable but still with the sequel of left-side hemiplegia.

Case 4

A one-month-old male child known to have multiple congenital anomalies, including complex congenital heart disease, due for cardiac repair, presented with a picture of neonatal sepsis. Blood culture grew CA-MRSA based on susceptibility, so vancomycin started. A chest radiograph was unremarkable, and transthoracic echocardiography showed large mass mobile in the right atrium close to the tricuspid valve with the possibility of vegetation with abscess. So, the patient was diagnosed with MRSA bacteremia complicated with native valve infective endocarditis. He underwent removal of tricuspid valve vegetations and abscess with tricuspid valve repair. Intraoperatively, swab was taken from the tricuspid valve for culture and histopathology and showed necrotic tissues with acute inflammatory exudate but the culture was negative,.However, he was presumed as a infective endocarditis case and continued on vancomycin for six weeks and rifampin for the initial two weeks. Bacteremia was eradicated smoothly with normal cardiac status at the end of therapy.

Case 5

**Figure 8:**
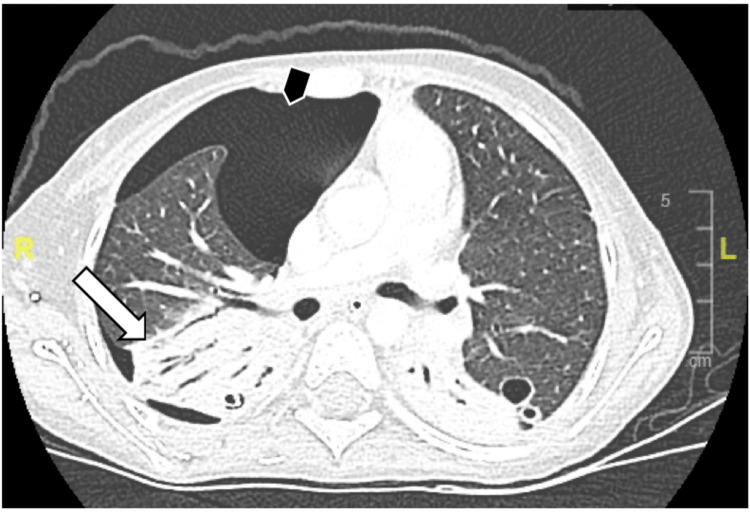
CT chest with contrast in Case 5 demonstrated patchy nodule in the left upper lobe and a small area of ground-glass opacity (white-long arrow), and large right pneumothorax (black-arrow).

A nine-year-old female child, who was a known patient of short bowel syndrome and total parenteral nutrition (TPN) dependent, was admitted to the critical care unit with septic shock and respiratory distress, for which she required inotropic and mechanical ventilation support. Initial workup showed elevated inflammatory markers, and blood culture from peripheral and central lines grew MRSA, for which the central line was removed. CT chest demonstrated a patchy nodule in the left upper lobe, a small area of ground-glass opacity, and large right pneumothorax with bilateral pleural effusion (Figure 8). Two chest tubes were inserted. Vancomycin and gentamicin were initiated, The patient was treated with intravenous vancomycin for six weeks and gentamicin for two weeks and showed significant clinical improvement.

Microbiological data

﻿*S. aureus* isolates were identified using standard methods. Methicillin resistance was defined by minimum inhibitory concentration using the BD Phoenix™ system (Becton, Dickinson and Company, New Jersey, United States) and confirmed with the E-test methods and interpretation guidelines of the Clinical and Laboratory Standards Institute (CLSI).

﻿All five MRSA isolates had similar antibiotic susceptibility patterns with resistance to oxacillin and susceptibility to ciprofloxacin, rifampin, vancomycin, trimethoprim-sulfamethoxazole, and linezolid. Three isolates had resistance to erythromycin with oxacillin. One isolate had resistance to erythromycin, clindamycin with oxacillin, and D-test positive. while one isolate had susceptibility to both erythromycin and clindamycin.

## Discussion

CA-MRSA becomes an emerging infection that could end with a devastating infection involving multiple organs with different consequences. Although there are previous similar studies of invasive CA-MRSA infection, however, the clinical diseases of the cases presented in this review are unique and worthy of being shared with other physicians in the field of pediatric and pediatric infectious diseases. 

All cases in this review did not end with mortality, but the infection had an impact on morbidity, like the 12-year-old girl with infective endocarditis which led to ischemic brain insult, ended with left hemiplegia. MRSA infection also led to some disabilities in the 11-year-old boy with advanced osteomyelitis associated with septic arthritis, in which the boy was not able to walk or use his leg initially, then he showed significant clinical and radiological improvement after around one month of appropriate treatment. However, he still required a walking aid to walk. As the infection causes significant destruction of the calcaneal bone with bone necrosis and shortening of the Achilles tendon, he will require plastic and orthopedics operations in the future.

Our series does not demonstrate specific risk factors for those patients to develop invasive MRSA infection, which is consistent with other previously reported studies. A study in 2007 in Taiwan studied 31 previously healthy children with invasive CA-MRSA infections and around ﻿93.5% had no risk factors [1]. Furthermore, a similar study done at the Aga Khan University Hospital, Karachi, Pakistan, reported five children with severe CA-MRSA infection, three below one year, and all of them had no risk factors [2]. 

In the third case of the present report, the patient was started empirically on ceftriaxone and vancomycin, which are the appropriate empiric antibiotic for suspected cases of meningitis, but when the initial workup came negative, vancomycin was discontinued and later she showed evidence of infective endocarditis with MRSA growth in the blood culture. Further studies are needed to look for risk factors for the development of severe-invasive CA-MRSA infection, so physicians can consider starting early empirical antibiotic coverage of MRSA in critically ill patients.

There are studies that assessed the antibiotic susceptibility pattern of CA-MRSA isolates [12]; however, a large study is needed to assess the susceptibility pattern of MRSA in different regions in Saudi Arabia that will guide empirical antibiotic coverage. 

In the neonate, the prevalence of MRSA infection is increasing with associated complications and high mortality rates [13], MRSA infection in neonates can disseminate and cause invasive infection as demonstrated in the second case in this report. MRSA infection needs to be considered in neonates with multi-focal disseminated osteomyelitis. Physicians such as pediatricians and neonatologists need to be aware of the complication of MRSA infection in all age groups including neonates and older children and to start appropriate antimicrobial regimens early on and involve specialists in infectious diseases in the management. 

In the absence of infectious diseases specialists, pediatricians need to be aware of and familiar with advanced technology such as ChatGPT (OpenAI Incorporated, San Francisco, California, United States) or telemedicine, which might support them in their medical decisions. Although these tools are still not so reliable, if they become more reliable and updated, they could support physicians who work in remote areas and lack support from specialized physicians in infectious diseases in choosing and starting appropriate empirical antibiotic coverage of MRSA in critically ill patients [14].

## Conclusions

Invasive MRSA infection in children is one of the serious infections that could lead to severe consequences. Five pediatric cases of MRSA invasive infection in Saudi Arabia were reported here with different infections like extreme sepsis, osteomyelitis, and infective endocarditis. All of them survived but they had significant morbidity. We emphasize the importance of pediatricians being aware of ﻿the broad spectrum of MRSA disease for early recognition and treatment. Appropriate empiric treatment for MRSA in children with suspected *S.aureus* infection is important to avoid and minimize the mortality and morbidity of this infection. The limitation of this report is that the number of cases is limited, and further studies with a larger number of CA-MRSA infections are needed to study the epidemiology of MRSA, the molecular characteristics of MRSA strain in the Saudi people group, and examine the risk factors to develop invasive MRSA infection.

## References

[1] Chen CJ, Su LH, Chiu CH, Lin TY, Wong KS, Chen YY, Huang YC (2007). Clinical features and molecular characteristics of invasive community-acquired methicillin-resistant Staphylococcus aureus infections in Taiwanese children. Diagn Microbiol Infect Dis.

[2] Khalid M, Junejo S, Mir F (2018). Invasive community acquired methicillin-resistant staphylococcal aureus (CA-MRSA) infections in children. J Coll Physicians Surg Pak.

[3] Iwamoto M, Mu Y, Lynfield R (2013). Trends in invasive methicillin-resistant Staphylococcus aureus infections. Pediatrics.

[4] Tsai YF, Ku YH (2012). Necrotizing pneumonia: a rare complication of pneumonia requiring special consideration. Curr Opin Pulm Med.

[5] Mermel LA, Allon M, Bouza E (2009). Clinical practice guidelines for the diagnosis and management of intravascular catheter-related infection: 2009 update by the Infectious Diseases Society of America. Clin Infect Dis.

[6] Thwaites GE, Edgeworth JD, Gkrania-Klotsas E (2011). Clinical management of Staphylococcus aureus bacteraemia. Lancet Infect Dis.

[7] Stankovic C, Mahajan PV (2006). Healthy children with invasive community-acquired methicillin-resistant Staphylococcus aureus infections. Pediatr Emerg Care.

[8] Bahrain M, Vasiliades M, Wolff M, Younus F (2006). Five cases of bacterial endocarditis after furunculosis and the ongoing saga of community-acquired methicillin-resistant Staphylococcus aureus infections. Scand J Infect Dis.

[9] Nourse C, Starr M, Munckhof W (2007). Community-acquired methicillin-resistant Staphylococcus aureus causes severe disseminated infection and deep venous thrombosis in children: literature review and recommendations for management. J Paediatr Child Health.

[10] Adam KM, Abomughaid MM (2018). Prevalence of methicillin-resistant Staphylococcus aureus in Saudi Arabia revisited: a meta-analysis. Open Public Health J.

[11] Alzomor O, Alfawaz T, Alshahrani D (2017). Invasive community-acquired methicillin-resistant Staphylococcus aureus (CA-MRSA) infection in children: case series and literature review. Int J Pediatr Adolesc Med.

[12] Vysakh PR, Jeya M (2013). A comparative analysis of community acquired and hospital acquired methicillin resistant Staphylococcus aureus. J Clin Diagn Res.

[13] Dolapo O, Dhanireddy R, Talati AJ (2014). Trends of Staphylococcus aureus bloodstream infections in a neonatal intensive care unit from 2000-2009. BMC Pediatr.

[14] Temsah O, Khan SA, Chaiah Y (2023). Overview of early ChatGPT’s presence in medical literature: insights from a hybrid literature review by ChatGPT and human experts. Cureus.

